# Books Reviewed

**Published:** 1868-12

**Authors:** 


### Editorial Department.

### Books Reviewed.

A Treatise on the Principles and Practice of Medicine, designed for the use of Practitioners and Students of Medicine. By Austin Flint. M. D., Professor of the Principles and Practice of Medicine in the Bellevue Hospital Medical College, Fellow of the New York Academy of Medicine, etc. Third edition, thoroughly revised. Philadelphia: Henry C. Lea, 1868.

It is now three years since the first appearance of Flint's Practice of Medicine, and each year has furnished us with a new edition of the work. The author informs us that: "since the publication of the second edition in December, 1866, much time has been devoted to its revision. Recognizing in the favor with which it has been received a proportionate obligation to strive constantly to increase its worthiness, the author has introduced in the present edition additions, derived from his clinical studies and from the latest contributions in medical literature, which it is believed will enhance considerably the practical utility of the work." It will thus be seen that the third edition is made to include all the recent advances in medicine which have been made during the past two years, and that every thing valuable within its scope is comprised in the present edition. We have on previous occasions spoken in detail of the merits of this work; and we do not propose to repeat what all of our readers have before this observed for themselves. However, we can never take up the work for reference upon any subject connected with the nature of disease, its progress, termination or treatment, without being additionally impressed with the faithfulness of description, accuracy of conclusion and completeness of discussion everywhere to be found within its pages. The things we most want to know are all expressed in such plain and convincing manner, with never a word too few or many, and with always an eye to the practical. Every sentence conveys an idea, and ideas which we supposed were old come up with such a freshness of connection and force of impression that they are really new. The whole range of practical medicine is comprised in a volume of about one thousand pages, and is so arranged as to be easily available for ready reference by the busy practitioner or for careful study by the student of medicine. It is *adapted* to the American profession, by which we mean that the author seems to have not only an acquired, but also an intuitive knowledge of our real wants. He does not dwell largely upon the speculative and possible, but established and well demonstrated truth he has presented in direct and forcible terms. Errors of the past or present he exposes with a faithful expression, never, however, unmindful of the opinions of others. His descriptions of disease are complete, and his views of treatment rational and correct.

A Hand-book of Vaccination. By Edward C. Seaton, M. D., Medical Inspector to the Privy Council, London. Philadelphia: J. B. Lippincott & Co., 1868.

This is a comprehensive and complete treatise upon vaccination; the main and important questions connected with it being considered in detail. The chapters upon vaccine or cow-pox, in the human subject; upon vaccinating and re-vaccination, and arrangements for the maintenance of lymph supply, are worthy of special attention. We do not know that any new facts are presented upon the general topics of vaccination, but the whole subject, as known, is embodied in its pages, and it will be gladly received by the profession as a standard work upon this subject.

---

The Science and Practice of Medicine. By William Aitkin, M. D., Edinburgh, Professor of Pathology in the Army Medical School. Second American from the fifth enlarged and carefully revised London edition; adopting the new Nomenclature of the Royal College of Physicians of London, with large additions by Meredith Clymer, M. D., ex-Professor of the Institutes and Practice of Medicine in the University of New York, etc. Philadelphia: Lindsay & Blakiston, 1868, 2 vols. 8vo.

On the receipt of the first volume of this work, we gave a somewhat detailed account of the improvements and additions made to this, the second American edition, and it is unnecessary to repeat, but we based our statements upon the author and editor's preface, and as some under-estimate of the additions made by the American editor were caused by the preface being printed while the second volume was passing through the press, we will again call attention to the additions made both by the author and editor.

The author informs us that he has spent fifteen months in the revision of his work, that the present edtition is increased over one hundred pages. The following subjects have been entirely re-written: Malignant Cholera, Paralysis, Epidemic Cerebro-Spinal Meningitis and Intestinal Obstruction. The subjects of Progressive Locomotor Ataxia, Progressive Muscular Atrophy, Glosso-laryngeal Paralysis, Aphasia and Dilitation of the Bronchial Tubes, the application of the Sphygmograph and its Tracings in Diseases where it has been of use, are subjects considered for the first time in this text-book.

Besides the large additions made to the author's text, *thirty-six* articles have been written by the editor, several of them on subjects now introduced for the first time in any text-book on the Prectice of Medicine:

1. Camp Measles.
2. Spinal Symptoms in Typhoid Fever.
3. Prognosis and Diagnosis of Typhoid Fever.
4. Chronic Malarial Toxæmia.
5. Pernicious Remittent Fever.
6. Typho-Malarial Fever.
7. Chronic Camp Dysentery.
8. Cholera Morbus.
9. Cholera Infantum.
10. Hereditary Syphilis.
11. Corpulence.
12. Gonorrhœal Rheumatism.
13. Delirium of Inanition.
14. Chronic Alcoholism.
15. Epidemic Cerebro-Spinal Meningitis
16. Progressive General Paralysis.
17. Acute Centripetal Paralysis.
18. Myo-Sclerosic Paralysis.
19. Physical Diagnosis of Diseases of the Cerebro-Spinal System.
20. Auscultation in Health and in Disease.
21. Irritable Heart.
22. Disease of the Heart, how far a Disqualification for Military Service?
23. Chronic Pyæmia.
24. Capillary Bronchitis.
25. Plastic Bronchitis.
26. Dilatation of the Bronchi.
27. Sclerosis of the Lung.
28. The Inoculation of Tubercle.
29. Curability of Consumption.
30. Acute and Rapid Phthisis.
31. The Neuroses of the Larynx.
32. Medication of the Throat and Lungs by Atomized Fluids.
33. Syphiloma of the Liver.
34. The Neuroses of the Stomach.
35. Addison's Keloid—Scleriasis.
36. Statistics of Tracheotomy.

This newly added material is essential to the perfection of the work, and brings it up to the present advance of medicine. The profession will peruse the newly written chapters with more interest than any other, since they are subjects of more recent and earnest inquiry.

We have repeatedly expressed our high estimate of the value and completeness of this treatise upon the science and practice of medicine, and could not add to our former expressions of approval.

---

The Medical Formulary; being a collection of Prescriptions derived from the Writings and Practice of many of the most Eminent Physicians in America and Europe, together with the usual Dietetic Preparations and Antidotes for Poisons; to which is added an Appendix, on the Endermic use of Medicines, and on the use of Ether and Chloroform. The whole accompanied with a few brief Pharmaceutical and Medical Observations, by Benjamin Ellis, M. D., late Professor of Materia Medica and Pharmacy in the Philadelphia College of Pharmacy. Twelfth edition, carefully revised and much improved, by Albert H. Smith, M. D., Fellow of the College of Physicians of Philadelphia; Lecturer on Obstetrics to the Philadelphia Lying-in Charity, etc. Philadelphia: Henry C. Lea, 1868.

This is a book which it is well enough to have, but to make any very great use of it indicates a fearful condition of superficiality. Physicians will, however, find in it correct forms of combination and prescription and imitation of accurate and well made prescriptions is better than copying incorrect and unscientific ones. The title-page, which we have copied, expresses most perfectly the character of the work, and there is no occasion for us to explain its scope and intention. For a work of the kind it is admirable, but we should hesitate to believe that any of our readers have much occasion for a medical formulary. We hope physicians will cultivate simplicity in form of prescription—will give one medicine at a time, so far as possible, and not confuse its operation by too many adjuncts. However, our author and editor have done their parts most admirably and the work is to be highly commended. While we deplore the *hand-books of medicine*, of all names and forms, we do it upon the general principle that "a little knowledge is a dangerous thing."

---

Outlines of Physiology, Human and Comparative. By John Marshall, F. R. S., Professor of Surgery in University College, London; Surgeon to the University College Hospital; with additions by Francis G. Smith, Professor of Institutes of Medicine in the University of Pennsylvania. Philadelphia: Henry C. Lea, 1868.

The limited time at our disposal, we have devoted to an examination of this work, and though at first we entertained the preconceived view that little could be gained by an American edition of any work upon Physiology, we now hold to the very opposite opinion. It has features which strongly demand recognition, and we regard it as a work upon physiology, both human and comparative, of great merit, one which will be chosen by teachers in schools and students in natural science as admirably adapted to their wants. The student of medicine will be attracted by it, as it presents a concise and comprehensive summary of modern physiological science, and the practitioner of medicine will be pleased with it, since every topic within the whole range of the science of life is noticed, to greater or less extent, as its importance demands. Upon the sciences of physiology, anatomy and chemistry depend most of what is positive in medicine. The progress and perfection of these has done much to rid us of long-cherished errors, and our future of progress and perfection in the knowledge and treatment of disease depends upon our attainments in chemistry and physiology. We all look earnestly in this direction for increased knowledge. We wait its developments before we announce our opinions as to the nature of disease or its rational modes of cure. Every physician, then, is interested to know what are the latest discoveries, what the results of the most recent experiments, what has been established as true or proved to be false. Physiology and chemistry constitute the foundation of rational medicine, and we hail every well directed effort to cultivate and extend these sciences. The work before us will be a popular text-book for the American profession, and, as we have before intimated, will be chosen in preference to others by many students and teachers engaged in scientific examinations and general literary pursuits. It contains a long list of illustrations, is elegantly and substantially bound, and is a valuable addition to our physiological literature.

---

The Opium Habit, with Suggestions as to the Remedy. New York: Harper & Brothers; 1868.

This is a popular and very readable book, with the following contents: Introduction; A successful attempt to abandon Opium; De Quincy's "Confessions of an English Opium-Eater; Opium Reminiscences of Coleridge; William Blair; Opium and Alcohol compared; Insanity and Suicide, from an attempt to abandon Morphine; A Morphine habit overcome; Robert Hall, John Randolph, William Wilberforce; What shall they do to be saved? Outlines of the Opium-Cure.

It will be seen at once what topics are introduced, and the manner of discussing them can be easily imagined, so that we shall not feel called upon to relate any facts contained in the work; it will, however, be found a very attractive book to those curious in watching the progress of a well told story. The main object of the work, aside from making itself very agreeable, is to furnish a warning to Opium-eaters, or those liable to become such, and the most graphic descriptions of the glories of the Opium-eater's dreams are contrasted with that world of torment, terror and torture which finally awaits those who cannot do anything to be saved.

---

A Treatise on Physiology and Hygiene, for Schools, Families and Colleges, by J. C. Dalton, M. D., Professor of Physiology in the College of Physicians and Surgeons, New York. With illustrations. New York: Harper & Brothers, publishers. London: Sampson Low, Son & Marston, 1868.

The name of the distinguished author is alone sufficient guarantee of the value of this work, and we have no doubt it will be received by schools, families and colleges, as a text-book better adapted to their wants than any other. We have examined it mainly with reference to its value as a book for schools and colleges and for the intelligent families of the country. Its style is at once attractive to every intelligent reader, and even the young students in our schools will appreciate and understand its teaching. It is written by a master hand, and how it could be made so perfect and at the same time so plain, is truly remarkable. For academies and schools, it is invaluable, and, indeed, for all students who desire to obtain concise and correct knowledge of the laws of life, and the conditions which prolong it. It is evidently designed for and adapted to schools and colleges, and it seems certain that its merits will be at once appreciated. It is illustrated wherever necessary to convey correct understanding, and the cuts are all executed in good style; the illustrations constitute an important and attractive feature of the work.

